# Cohort study with 4‐year follow‐up of myopia and refractive parameters in primary schoolchildren in Baoshan District, Shanghai

**DOI:** 10.1111/ceo.13195

**Published:** 2018-04-16

**Authors:** Yingyan Ma, Haidong Zou, Senlin Lin, Xun Xu, Rong Zhao, Lina Lu, Huijuan Zhao, Qiangqiang Li, Ling Wang, Jianfeng Zhu, Xiangui He

**Affiliations:** ^1^ Department of Preventative Ophthalmology Shanghai Eye Disease Prevention and Treatment Center, Shanghai Eye Hospital Shanghai China; ^2^ Department of Ophthalmology Shanghai General Hospital, Shanghai Jiao Tong University, Shanghai Key Laboratory of Ocular Fundus Diseases, Shanghai Engineering Center for Visual Science and Photomedicine Shanghai China; ^3^ Shanghai Shenkang Hospital Development Center Shanghai China; ^4^ Baoshan Center for Disease Prevention and Control Shanghai China; ^5^ Department of Maternal and Child Health School of Public Health, Key Laboratory of Public Health Safety, Ministry of Education, Fudan University Shanghai China

**Keywords:** axial length, myopia, progression, schoolchildren, spherical equivalent refraction

## Abstract

**Importance:**

Cohort studies could not only reveal associations between change of refractive components and onset/progression of myopia, but also risk factors, which is important for understanding mechanism and providing strategies.

**Background:**

Prevalence of myopia is high in Shanghai, being reported to be 52.2% in children aged 10 years old.

**Design:**

Cohort study.

**Participants:**

A total of 1856 students from six randomly selected primary schools in Baoshan District, Shanghai.

**Methods:**

Children underwent comprehensive ocular measurement, including axial length (AL), corneal curvature radius and cycloplegic auto‐refraction. Questionnaires about eye usage time were collected. Grade 1 students were followed for 4 years, and grade 2 and 3 students for 2 years.

**Main Outcome Measures:**

(i) Change of spherical equivalent (SE) and AL and (ii) risk factors for progression and incidence of myopia.

**Results:**

The average 2‐year progress of SE was 0.91D, 0.91D and 1.11D for grade 1, 2 and 3, respectively, and the average elongation of AL was 0.70 mm, 0.64 mm and 0.71 mm, respectively. Only parental myopia, but not near work time, near work diopter time, outdoor activity time or attending tutoring classes, was associated with myopia incidence and progression in the present population. Using baseline SE could be a simple and effective indicator for myopia prediction.

**Conclusions and Relevance:**

Incidence and progression of myopia is relatively high in schoolchildren in Shanghai compared with children of Western countries, East Asia and other parts of China. Effective strategies to control myopia prevalence are in urgent need.

## Introduction

In 2000, there were 1.41 billion people with myopia (spherical equivalent [SE] ≤−0.5D) globally, and among them 0.16 billion with high myopia (SE ≤ −5.0D). It is estimated that up to 2050, there will be 4.76 billion people with myopia and 938 million with high myopia, accounting for 50% and 10% of the world population.^1^ Myopia can impair vision if not fully corrected, and high myopia carries an increased risk of several blinding diseases such as glaucoma, retinal detachment and myopic retinopathy.^2^ As a result, myopic retinopathy has become one of the leading causes of irreversible visual impairment and blindness among adult population in many regions of the world.^3^, ^4^, ^5^, ^6^

The prevalence of myopia is especially high for East‐Asian children, as reported in our previous study, the prevalence was 52.2% in the 10‐year‐old schoolchildren in Shanghai, China.^7^ Therefore, finding risk factors of myopia for children is especially important. Cohort studies such as Singapore Cohort Study of the Risk Factors for Myopia, Orinda Longitudinal Study of Myopia, Collaborative Longitudinal Evaluation of Ethnicity and Refractive Error, Sydney Myopia Study, Guangzhou Twins Eye Study and many others provided evidence to understand possible risk factors of myopia, such as parental myopia, near work and outdoor activity.^8^, ^9^, ^10^, ^11^, ^12^, ^13^ However, in China, with a high prevalence of myopia in teenagers,^14^ there are limited number of cohort studies with relatively long follow‐up in young schoolchildren.

Guo *et al*. followed 643 primary schoolchildren for 4 years, and found that an increase in myopia is associated with parental myopia, less time outdoors and more time indoors.^15^, ^16^ Another study in Beijing also reported 1‐year cohort in primary schoolchildren, and suggested that shorter distance from near work and shorter time outdoors were associated with an increase in incident myopia.^17^ Both of the two studies measured non‐cycloplegic refraction data, which will be inaccurate in evaluating young children's refractive status.^18^ Other studies, such as the Guangzhou Twins Eye Study and the Beijing Myopia Progression Study,^13^, ^19^ measuring cycloplegic refraction, suggested risk factors such as long near work time, parental myopia and short outdoor time were associated with myopia; however, the Beijing Myopia Progression Study did not observe relationship between outdoor and myopia.^19^ In another study performed by our study group in Jiading District, Shanghai, the 1‐year cohort results suggested that in addition to time outdoor and near work, near work related behaviours were also associated with incident myopia in schoolchildren.^20^ The present study followed primary schoolchildren for 4 years (from 2010 to 2014) in Baoshan District, another suburb area in Shanghai, and measured their cycloplegic refractive parameters aiming to describe incidence of myopia, change of refraction and refractive components, to explore possible risk factors for myopia incidence and to try to building models to predict myopia incidence.

## Methods

### Study population, inclusion and exclusion criteria

This study is a 4‐year school‐based cohort study in Baoshan District, Shanghai. Baoshan District is located in the north of Shanghai, one of the biggest cities in China. The district has a population of 1 935 000 regular residents, and the number of students in primary schools was 61 740. The enrolment rate for compulsory education in primary schools was 100% in 2011 in Baoshan. The prevalence of myopia in primary schools in Baoshan increased significantly from 11.3% in children of 7 years old to 52.9% in children of 11 year old,^21^ which is similar to what was reported in children of same age in urban Guangzhou and in Shandong province, China.^22^, ^23^

Stratified random sampling was used to select six primary schools in Baoshan District, Shanghai. Among them, children of grades 1–3 were included in the study. Considering the time span of primary schooling in Shanghai, children of grades 2 and 3 were followed for 2 years, and children of grade 1 were followed for 4 years. The investigation was carried out every 2 years after baseline examinations. Exclusion criteria were children with severe ocular diseases other than refractive error, such as cataract, children who are not cooperative with the examinations and children unable to follow up in the study. The study was approved by the Shanghai General Hospital Ethics Committee and adhered to the tenets of the Declaration of Helsinki. All the participants gave their written informed consent from their parents or guardians.

### Field investigations and questionnaires

The first visit was conducted from May 2010 to April 2011, the second visit was conducted from May 2012 to April 2013 and the third visit was conducted from May 2014 to April 2015. The examination order for the six schools was the same during the two visits, in order to ensure the 2‐year gap between the visits. All the examinations were performed during weekdays while the children were in school. One ophthalmologist, five optometrists and two public health doctors conducted the examinations. Children underwent uncorrected visual acuity (Standard Logarithmic Visual Acuity E Chart, 5 m), slit lamp examination, intraocular pressure by non‐contact tonometer, cycloplegia, cycloplegic auto‐refraction and corneal curvature radius (CR) by a table‐mounted auto‐refractor (KR‐8800, Topcon, Tokyo, Japan), axial length (AL) by an IOLMaster (version 5.02, Carl Zeiss Meditec, Oberkochen, Germany) and best‐corrected visual acuity. Cycloplegia was induced by instillation of one drop of 0.5% tropicamide every 5 min five times. When the pupil was dilated to 6 mm or greater in the absence of a light reflex, cycloplegia was considered complete. Auto‐refraction was measured at an average of 20 min after the last drop of tropicamide. The ophthalmologist examined children under a slit lamp and determined whether a child was suitable for cycloplegia and whether the cycloplegia was complete. Only those with full cycloplegia were included in the study. The optometrists measured non‐contact intraocular pressure, auto‐refraction, AL and CR. Any children with uncorrected visual acuity lower than 20/25 in either eye was given subjective optometry to obtain the best‐corrected visual acuity. If severe ocular diseases were detected at the screening, we sent the examination results and our suggestions to the schools, and the school health teachers gave the eye examination reports to the children‘s parents. Those children were subsidized for costs of examinations if they went to the hospitals designated by the study.

Myopia risk factor related questionnaires were filled by children with the help of their parents at baseline. The questionnaires collected parental myopia and average eye usage time at home or after school in weekdays and weekends during the most recent month. Eye usage time were investigated for time spent on reading books/magazines and writing homework, watching television, using computer, playing electronic devices such as mobile phone, tablet computer and video games, doing outdoor activities and attending tutoring classes. Information about parental myopia was also included in the questionnaires.

### Statistical analyses

The SE was calculated as sphere power + 0.5 × cylinder power. Myopia was defined as SE ≤−0.5D in the right eye. The right eye was chosen for data analyses, because the SE refraction in the right and the left eye are highly correlated with each other at baseline (Spearman correlation coefficient = 0.913, *P* < 0.001), and at the second visit (Spearman correlation coefficient = 0.926, *P* < 0.001).

The spherical diopter, cylinder diopter, CR and AL were recorded at baseline and the follow‐ups. Two‐year change in refractive status and ocular components was defined as: measurement at baseline (2010–2011) − measurement at the second visit (2012–2013). Four‐year change in refractive status and ocular components was defined as: measurement at baseline (2010–2011) − measurement at the third visit (2014–2015). The 2‐year/4‐year incident myopia was defined as being non‐myopic at baseline and being myopic at the second/third visit in the right eye. As we carried out the examinations every 2 years, we presented the incidence of myopia, change of refraction and associated factors in the first 2‐year follow‐up for children from grade 1 to grade 3. Only in the analyses of predicting myopia incidence, we included the 4‐year cohort, because predicting myopia in a relatively long time would be of great value.

Eye usage time was classified into three categories as low, moderate, and high according to baseline population tertiles. The near work time per week was calculated as the total amount of time including reading books/magazines and writing homework, watching television, using computer and playing electronic devices such as mobile phone, tablet computer and video games in the whole week (5 × time in weekdays + 2 × time in weekends). Diopter hours of near work per week were also investigated, calculated as 3 × reading books/magazines and writing homework + 2 × using computer and playing electronic devices such as mobile phone, tablet computer and video games + 1 × watching television.^11^ The tertile ranges for time spent in near work per week were low (≤26.5 h), moderate (≥27 to <37.4 h) and high (≥37.5 h). The tertile ranges for diopter hours spent in near work per week were low (≤63.5 h), moderate (≥ 64 to <87.5 h) and high (≥88.0 h). Tertile ranges for time spent outdoors per week were low (<4 h), moderate (≥4 to <9 h) and high (≥9 h).

Multivariate logistic regression analyses were performed to investigate the associations between eye usage time and risk of myopia adjusted for age, gender, parental myopia and baseline SE. In those who were not myopic at baseline, multiple linear regression analyses were made to explore associated factors for the progression of SE refraction. To test whether baseline SE, AL or AL/CR could predict the incidence of myopia, receiver‐operating characteristic (ROC) curves were plotted to calculate area under the curve (AUC) and best cut‐off values. SPSS 22.0 (IBM SPSS Inc., Chicago, IL, USA), SAS 9.4 (SAS Institute, Cary, NC, USA) and MedCalc 11.4.2.0 (MedCalc Software, Ostend, Belgium) were used for statistical analyses. A *P* value of less than 0.05 was considered statistically significant.

## Results

### Change of refractive parameters and ocular components

**Figure 1:**
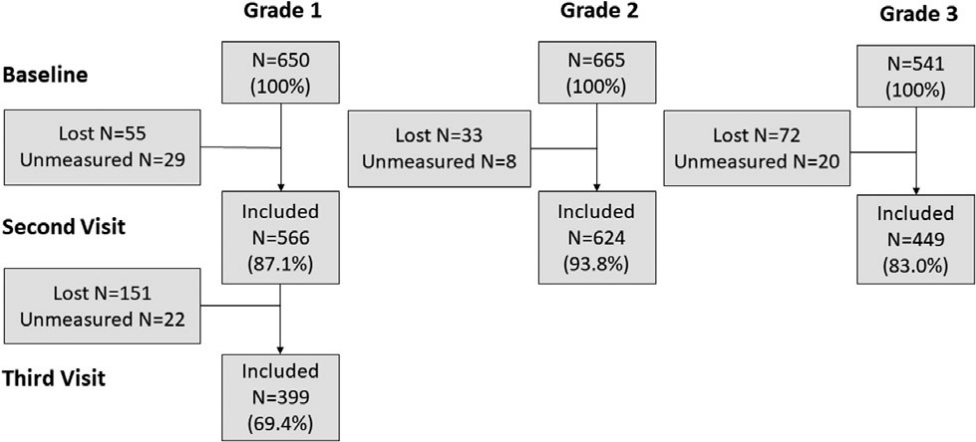
Flow‐chart for the inclusion and exclusion of the study population.

At baseline 1856 children of grades 1–3 completed the ocular examinations, among those who were not myopic at baseline (*n* = 1567), a number of 1385 (88.4%) attended the refraction measurement at the second visit. The characteristics for children who were included in the baseline (*n* = 1856) were presented in Table 1. Among those who attended the second visit, 69.4% grade 1 children were examined at the third visit. The flow chart of the included study population was shown in Figure 1. In the 2‐year follow‐up, comparisons between those followed up and those who were lost showed no significant difference in baseline refractive parameters such as SE and AL with *P* values of 0.504 and 0.107, respectively. In the 4‐year follow‐up, the differences were not statistically significant as well (with *P* value of 0.09 and 0.183 for baseline SE and AL).

**Table 1:** Characteristics of children who were included in the study at baseline (*n* = 1856)

	Grade 1	Grade 2	Grade 3	All	*P* value†
Age	7.1 ± 0.6	8.1 ± 0.6	9.2 ± 0.8	8.1 ± 1.1	<0.001
Gender, no. of girls (%)	307 (47.2)	308 (46.3)	268 (49.5)	883 (47.6)	0.525
Prevalence, *n* (%)	48 (7.4)	101 (15.2)	140 (25.9)	289 (15.6)	<0.001
SE (D)	0.64 ± 0.97	0.33 ± 1.22	0.02 ± 1.29	0.35 ± 1.19	<0.001
AL (mm)	22.84 ± 0.72	23.11 ± 0.82	23.28 ± 0.89	23.06 ± 0.83	<0.001
CR (mm)	7.86 ± 0.26	7.87 ± 0.27	7.85 ± 0.25	7.86 ± 0.26	0.368

† Comparisons of characteristics among grades 1–3 students using one‐way analysis of variance for continuous variables, and using chi‐square test for categorical variables.

AL, axial length; CR, corneal curvature radius; SE, spherical equivalent.

For those were followed in the second visit (*n* = 1639), the average age at baseline was 8.1 (standard deviation [SD] = 1.0), with 844 being boys (51.5%). Among those who were not myopic at baseline (*n* = 1385), a total of 501 (36.2%) children became myopia (SE ≤ −0.5D) in 2 years. The proportion (number of newly developed myopic children in 2 years/number of children at baseline) was 30.0% (170/566), 29.2% (182/624) and 33.2% (149/449) for grades 1, 2 and 3 students. The average 2‐year change of SE is 0.97D (95% confidence interval [CI]: 0.92–1.01) and AL is 0.68 mm (0.66–0.70) for all the children measured.

To present the 2‐year change of refraction and refractive components, children were classified into three categories: non‐myopia as SE >−0.5D at both visits, incipient myopia as SE >−0.5D at baseline and SE ≤−0.5D at the second visit and persistent myopia as SE ≤−0.5D at baseline (Table 2). The average progress of SE was 0.40D (0.36–0.44), 1.57D (1.51–1.63) and 1.76D (1.65–1.87) for non‐myopia, incipient myopia and persistent myopia. Accordingly, the average increase of AL was 0.48 mm (0.46–0.50), 0.90 mm (0.86–0.93) and 0.96 mm (0.91–1.00). Therefore, an increase of a millimeter in AL corresponded to SE progression of 0.83D, 1.74D and 1.83D for non‐myopia, incipient myopia and persistent myopia. Significant differences were observed in non‐myopia, incipient myopia and persistent myopia for change of refraction and refractive components, except for change of CR in grades 1 and 2 students (Table 2).

**Table 2:** Two‐year progression of myopia and changes in ocular biometry in the schoolchildren (*n* = 1639)

	Grade 1	Grade 2	Grade 3	*P* value†
Change SE, D				
Non‐myopia	0.40 (*n* = 355)	0.41 (*n* = 350)	0.38 (*n* = 179)	0.8475
	0.34 to 0.46	0.35 to 0.46	0.30 to 0.46	
Incipient myopia	1.70 (*n* = 170)	1.54 (*n* = 182)	1.44 (*n* = 149)	0.0033
	1.58 to 1.81	1.45 to 1.64	1.34 to 1.54	
Persistent myopia	2.05 (*n* = 41)	1.59 (*n* = 92)	1.78 (*n* = 121)	0.0203
	1.76 to 2.34	1.37 to 1.82	1.66 to 1.91	
All	0.91 (*n* = 566)	0.91 (*n* = 624)	1.11 (*n* = 449)	<0.001
	0.83 to 0.99	0.84 to 0.98	1.03 to 1.19	
*P* value‡	<0.0001	<0.0001	<0.0001	
Change DC, D				
Non‐myopia	−0.06 (*n* = 355)	−0.02 (*n* = 350)	−0.02 (*n* = 179)	0.2616
	−0.09 to −0.02	−0.05 to 0.01	−0.07 to 0.03	
Incipient myopia	0.07 (*n* = 170)	0.02 (*n* = 182)	0.09 (*n* = 149)	0.1690
	0.01 to 0.12	−0.04 to 0.08	0.04 to 0.15	
Persistent myopia	0.05 (*n* = 41)	0.11 (*n* = 92)	0.16 (*n* = 121)	0.3718
	−0.07 to 0.16	−0.01 to 0.23	0.09 to 0.24	
All	−0.02 (*n* = 566)	0.01 (*n* = 624)	0.07 (*n* = 449)	0.002
	−0.05 to 0.01	−0.02 to 0.04	0.03 to 0.10	
*P* value‡	0.0008	0.0156	<0.0001	
Change AL, mm				
Non‐myopia	−0.50 (*n* = 350)	−0.45 (*n* = 348)	−0.49 (*n* = 176)	0.1101
	−0.52 to −0.47	−0.49 to −0.42	−0.55 to −0.44	
Incipient myopia	−1.03 (*n* = 168)	−0.86 (*n* = 181)	−0.79 (*n* = 149)	<0.0001
	−1.08 to −0.98	−0.92 to −0.80	−0.84 to −0.73	
Persistent myopia	−1.11 (*n* = 41)	−0.91 (*n* = 92)	−0.94 (*n* = 120)	0.0110
	−1.21 to −1.01	−0.98 to −0.83	−1.02 to −0.87	
All	−0.70 (*n* = 559)	−0.64 (*n* = 621)	−0.71 (*n* = 445)	0.004
	−0.73 to −0.67	−0.67 to −0.61	−0.75 to −0.67	
*P* value‡	<0.0001	<0.0001	<0.0001	
Change CR, mm				
Non‐myopia	−0.01 (*n* = 353)	0.00 (*n* = 347)	−0.01 (*n* = 176)	0.3507
	−0.02 to 0.00	−0.01 to 0.01	−0.02 to 0.00	
Incipient myopia	−0.01 (*n* = 168)	0.01 (*n* = 181)	0.00 (*n* = 149)	0.1381
	−0.01 to 0.00	0.00 to 0.02	−0.01 to 0.01	
Persistent myopia	−0.02 (*n* = 41)	−0.01 (*n* = 92)	0.01 (*n* = 121)	0.0197
	−0.04 to −0.01	−0.04 to 0.01	0.00 to 0.02	
All	−0.01 (n = 562)	0.00 (*n* = 620)	0.00 (*n* = 444)	0.157
	−0.14 to 0.00	−0.01 to 0.01	−0.01 to 0.00	
*P* value‡	0.2391	0.1999	0.0162	
Change SE/Change AL (D/mm)				
Non‐myopia	−0.80	−0.91	−0.78	
Incipient myopia	−1.65	−1.79	−1.82	
Persistent myopia	−1.85	−1.75	−1.89	
All	−1.30	−1.42	−1.56	

† Comparisons of change of refraction and refractive components among grades 1–3 students using one‐way analysis of variance.

‡ Comparisons of change of refraction and refractive components among non‐myopia, incipient myopia and persistent myopia using one‐way analysis of variance.

AL, axial length; CR, corneal curvature radius; DC, diopter of cylinder power; SE, spherical equivalent.

As grade level increased, the change of SE and AL decreased for incipient myopia and persistent myopia, but not for non‐myopia. No change was observed for progression of cylinder diopter as grade level increased. For the change of CR, significant change was presented in the persistent myopia as grade level changed. For persistent myopia, grade 1 children showed an increase in CR; however, grade 3 children presented a decrease in CR (Table 2).

### Associated risk factors of myopia

Among those who were not myopic at baseline (*n* = 1385), a total of 1184 (85.5%) students had fulfil the questionnaires with the help of their parents. There are no statistical differences between those had accomplished the questionnaires and those who had not in age, gender, baseline SE and AL (all *P* > 0.05).

**Figure 2:**
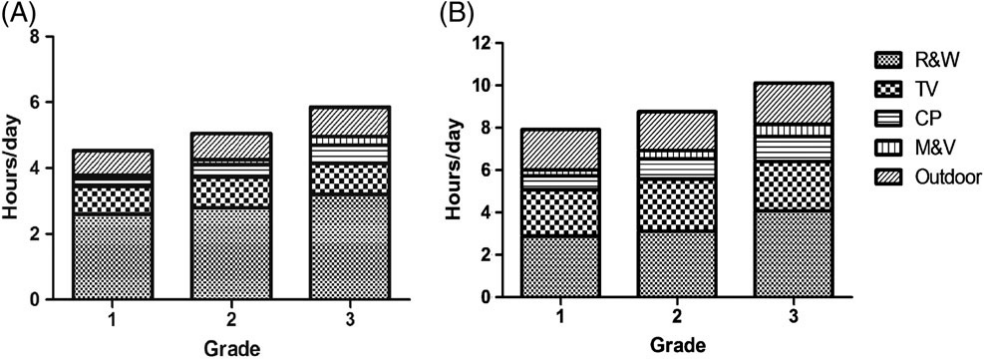
Average hours per day of various kinds of activities at weekdays (a) and weekends (b). CP, using computer; M&V, playing electronic devices such as mobile phone, tablet computer, and video games; Outdoor, outdoor activities; R&W, reading and writing; TV, watching television.

Figure 2 displayed the average hours per day of various kinds of activities at weekdays and weekend. For the whole population, the average time spent on reading and writing was 3.28 (SD = 1.97) h/day and 2.82 (SD = 1.48) h/day at weekdays after school and at weekend. However, the average time spent on outdoor was 0.82 (SD = 0.98) h/day and 1.89 (SD = 1.61) h/day at weekdays after school and at weekend. Time of reading and writing increased steadily from 2.60 h/day to 3.20 h/day at weekdays and from 2.88 h/day to 4.08 h/day at weekend as grade level increases (both *P* < 0.001). Time of watching television, although constituting a quite large proportion of daily activity did not change much with grade level at weekdays (*P* = 0.368). Time of playing computer, mobile phone, tablet and video games also increased as grade level increases (all *P* < 0.001). Time of tutoring classes per week was greater in grades 2 and 3 than in grade 1, but not statistically significant (*P* = 0.081). On the contrary, outdoor time did not change much while grade level increases at weekdays and weekend (*P* = 0.178 and 0.558). The average time spent outdoor was 0.76 h/day, 0.81 h/day and 0.89 h/day at weekdays for children of grades 1, 2 and 3. And the outdoor time at weekend was 1.91 h/day, 1.82 h/day and 1.95 h/day for each grade. The questionnaires also reflected that although children spent less time on reading and writing at weekend, they increased the time for other near work activities such as watching television and playing computer, instead of going outdoors for sports or other activities.

Logistic analyses showed that the 2‐year incident myopia was only associated with parental myopia, but not with time of near work, diopter hours of near work, time of outdoor activities or attending tutoring classes (Table 3). The results were similar for the associations between those possible risk factors and progression of SE during the 2 years (Table 4). Although incidence of myopia (also the progression of SE) increased with number of parents in all grades, the effect of parental myopia was stronger in lower grade (grade 1) than in senior grade (grades 2 and 3). The results were similar in the 4 year follow‐up cohort (data not shown). Only parental myopia (two myopic parents compared with no myopic parent) was associated with progression of SE, with the beta coefficient of 0.79 (95% CI: 0.33–1.25, *P* = 0.001) after adjusted for age, gender and baseline SE.

**Table 3:** Associations between risk factors and incident myopia (*n* = 1385)† ^,^ ‡

Grade		1		2		3		All	
		Incident myopia, %	OR (95% CI)	Incident myopia, %	OR (95% CI)	Incident myopia, %	OR (95% CI)	Incident myopia, %	OR (95% CI)
Parental myopia	0	29.5	Ref	32.7	Ref	44.7	Ref	34.3	Ref
	1	32.6	1.03 (0.56–1.91)	38.2	0.85 (0.43–1.69)	44.6	0.92 (0.42–2.01)	37.6	0.96 (0.65–1.41)
	2	55.1	3.02 (1.41–6.50)**	50.0	0.87 (0.26–2.85)	53.6	2.46 (0.89–6.80)	53.5	2.28 (1.33–3.91)**
Near work	L	33.0	Ref	35.1	Ref	44.3	Ref	35.5	Ref
	M	34.0	1.12 (0.64–1.96)	34.8	1.18 (0.58–2.36)	47.0	1.41 (0.61–3.29)	37.6	1.18 (0.81–1.72)
	H	28.4	0.92 (0.48–1.77)	34.8	1.29 (0.64–2.59)	47.0	1.01 (0.47–2.20)	37.8	1.11 (0.75–1.63)
Near work diopter	L	33.7	Ref	35.2	Ref	50.0	Ref	36.8	Ref
	M	31.2	0.99 (0.57–1.74)	36.3	0.99 (0.49–1.97)	45.5	0.73 (0.30–1.75)	36.3	0.98 (0.67–1.43)
	H	31.4	0.93 (0.49–1.78)	33.1	1.09 (0.55–2.16)	45.7	0.67 (0.31–1.45)	37.8	1.00 (0.68–1.46)
Outdoor	L	33.6	1.23 (0.66–2.32)	38.0	1.19 (0.61–2.34)	44.4	0.90 (0.44–1.85)	37.9	1.12 (0.77–1.64)
	M	28.4	0.83 (0.47–1.46)	33.1	0.79 (0.40–1.55)	39.6	0.77 (0.39–1.54)	32.9	0.82 (0.57–1.18)
	H	35.9	Ref	36.1	Ref	53.7	Ref	41.0	Ref
Tutoring class	0	33.4	Ref	35.5	Ref	43.4	Ref	36.4	Ref
	1	28.4	0.67 (0.36–1.22)	34.1	1.18 (0.63–2.20)	50.8	1.33 (0.73–2.41)	38.2	1.09 (0.78–1.53)

* *P* < 0.05,

** *P* < 0.01,

*** *P* < 0.001.

† The odds ratios (ORs) were calculated using logistic regression analysis and were adjusted for age, gender, baseline spherical equivalent refraction and parental myopia. Parental myopia analyses were only adjusted for age, gender and baseline spherical equivalent refraction.

‡ L, M, and H stands for the tertile ranges (low, moderate and high) for time spent in near work, diopter of near work and outdoor activity, which could be referred in methods.

For tutoring class, it was classified into 1 and 0, which stands for having attended tutoring class or not.

CI, confidence interval.

**Table 4:** Associations between baseline risk factors and progress of spherical equivalent refraction (*n* = 1385)† ^,^ ‡

Grade		1		2		3		All	
		Beta coefficients	95% CI	Beta coefficients	95% CI	Beta coefficients	95% CI	Beta coefficients	95% CI
Parental myopia	0	Ref		Ref		Ref		Ref	
	1	0.09	−0.12‐0.29	0.05	−0.15‐0.25	0.12	−0.11‐0.34	0.08	−0.04‐0.20
	2	0.53***	−0.28‐0.79	0.23	−0.10‐0.56	0.34*	0.04–0.64	0.41***	0.24–0.57
Near work	L	Ref		Ref		Ref		Ref	
	M	−0.05	−0.24‐0.13	−0.01	−0.21‐0.18	−0.09	−0.34‐0.16	−0.05	−0.17‐0.06
	H	−0.12	−0.33‐0.09	−0.03	0.23–0.16	−0.11	−0.35‐0.13	−0.08	−0.20‐0.04
Near work diopter	L	Ref		Ref		Ref		Ref	
	M	−0.10	−0.28‐0.08	−0.04	−0.23‐0.15	−0.11	−0.37‐0.15	−0.78	−0.19‐0.04
	H	−0.13	−0.34‐0.08	−0.11	−0.30‐0.08	−0.11	−0.34‐0.13	−0.10	−0.22‐0.02
Outdoor	L	0.13	−0.07‐0.33	−0.13	−0.32‐0.06	0.05	−0.17‐0.27	0.02	−0.10‐0.13
	M	−0.12	−0.31‐0.07	−0.05	−0.23‐0.14	−0.02	−0.23‐0.19	−0.06	−0.17‐0.05
	H	Ref		Ref		Ref		Ref	
Tutoring class	0	Ref		Ref		Ref		Ref	
	1	−0.03	−0.21‐0.16	−0.04	−0.21‐0.13	0.06	−0.12‐0.24	0.02	−0.09‐0.12

* *P* < 0.05,

** *P* < 0.01,

*** *P* < 0.001.

† The beta coefficients were calculated using linear regression analysis and were adjusted for age, gender, baseline spherical equivalent refraction and parental myopia. Parental myopia analyses were only adjusted for age, gender and baseline spherical equivalent refraction.

‡ L, M, and H stands for the tertile ranges (low, moderate and high) for time spent in near work, diopter of near work and outdoor activity, which could be referred in methods.

For tutoring class, it was classified into 1 and 0, which stands for having attended tutoring class or not.

CI, confidence interval.

### Baseline refraction and prediction of myopia

**Figure 3:**
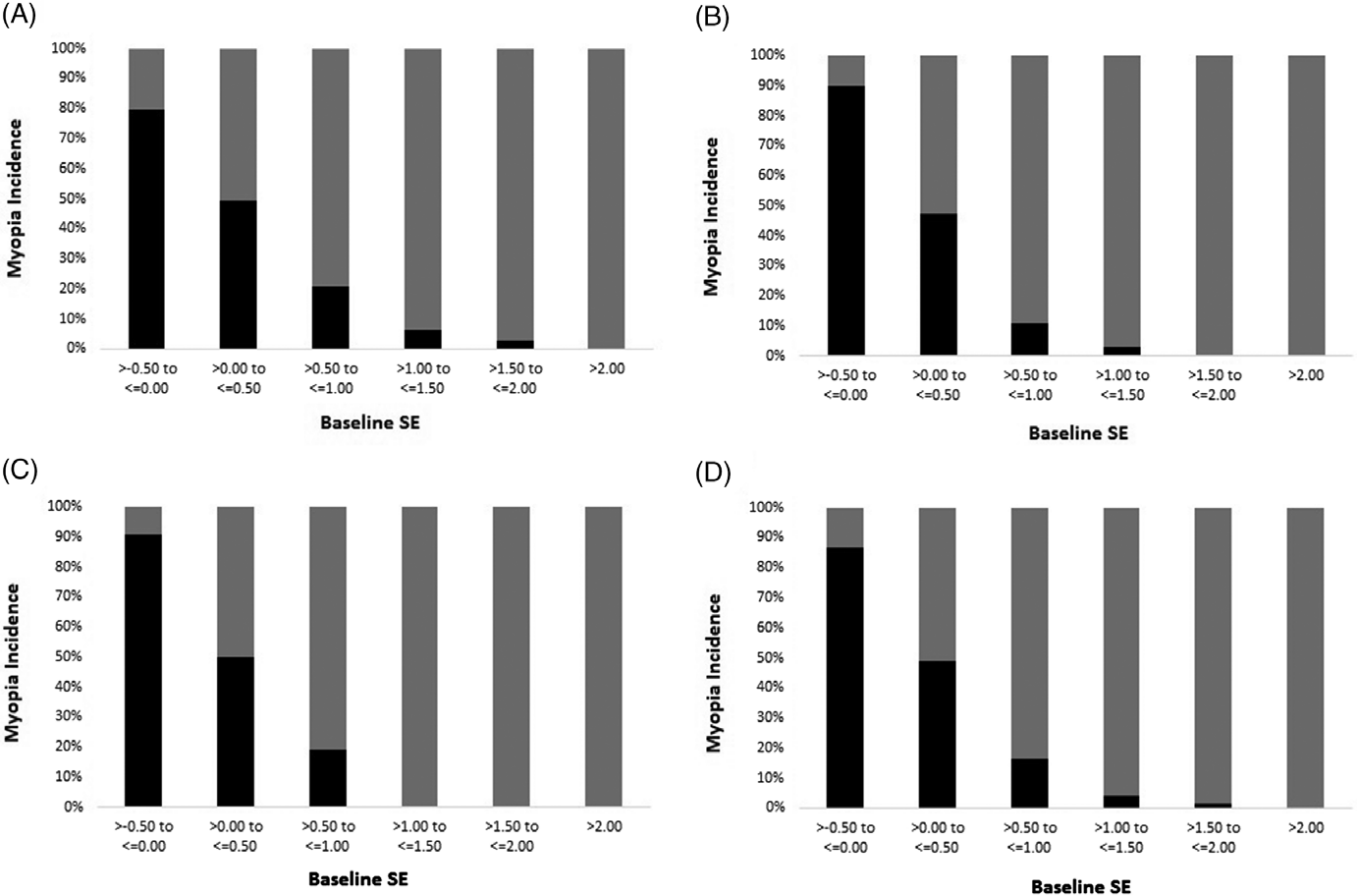
Incidence of myopia according to baseline spherical equivalent (SE) refraction for grade 1 (a), grade 2 (b), grade 3 (c) and the whole population (d).

For the whole population, incidence of myopia decreases as the SE of baseline refraction increased (more hyperopia less incidence of myopia), from 86.8% in the baseline SE ≤0.00D to 0% in the SE >2.00D. For grade 1 students, even if the SE was more hyperopic than 1.0D at baseline, there were still certain chances to be myopic in 2 years (about 9%); however, for students of grade 2 or 3, the possibility of being myopic was extremely lower when the baseline SE was greater than 1D (Fig. 3). As children grow older, less were remained in the more hyperopic groups. However, those who remained in the hyperopic groups were less likely to be myopic.

**Figure 4:**
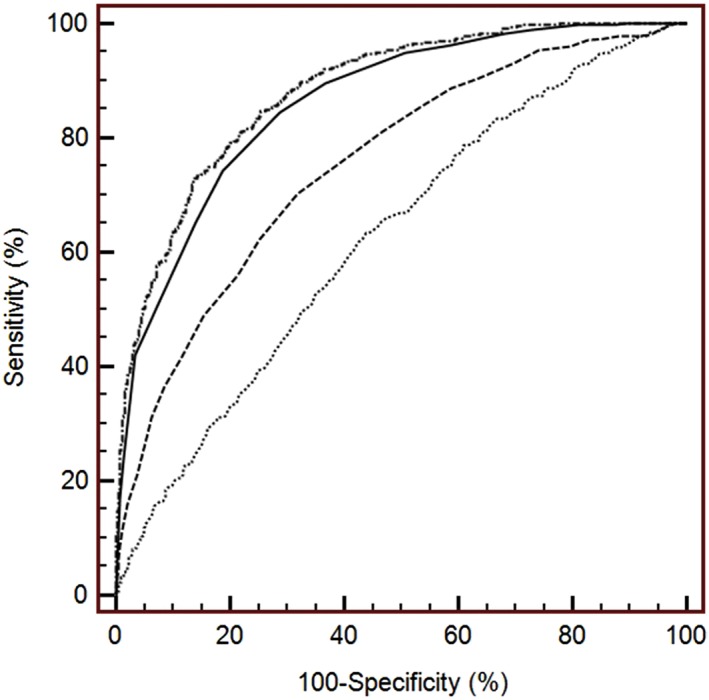
Receiver operating characteristic (ROC) curves analyses for predicting 2‐year incidence myopia using baseline axial length (AL) (dotted line), AL/corneal curvature radius (CR) (dash line), spherical equivalent (SE) refraction (solid line) and a combination of SE, AL/CR, age, gender and parental myopia (dotted‐dash line).

Using baseline AL, AL/CR and SE to predict 2‐year incident myopia achieved AUCs of 0.626 (95% CI: 0.600–0.652), 0.755 (0.731–0.778) and 0.862 (0.842–0.879) (Fig. 4). Combining baseline SE, AL/CR, age, gender and parental myopia, the AUC increased to 0.880 (0.861–0.898) by 0.018 compared with using baseline SE alone for prediction (Fig. 4). Additional analyses were made according to grade level and results were shown in Table 5. The ROC curve showed that the best cut‐off (the maximum value of the sum of sensitivity and specificity) for prediction of 2‐year incident myopia was SE ≤ 0.5D, with sensitivity and specificity value of 84.6% and 71.0%. If the criterion of 80% for specificity was set, the SE was ≤0.375D to predict myopia incidence, with sensitivity and specificity of 74.5% and 81.1%. The analyses for separate grades were shown in Table 5.

**Table 5:** Receiver‐operating characteristic curve analyses results for the 2‐year prediction of myopia according to different grade levels

	Grade 1	Grade 2	Grade 3
AUC for different prediction methods			
AL alone	0.567 (0.515–0.619)	0.616 (0.566–0.665)	0.700 (0.643–0.756)
AL/CR alone	0.745 (0.699–0.791)	0.724 (0.680–0.769)	0.798 (0.750–0.846)
Combination	0.858 (0.825–0.891)	0.887 (0.860–0.915)	0.894 (0.861–0.927)
SE alone	0.839 (0.804–0.874)	0.882 (0.853–0.910)	0.857 (0.819–0.896)
SE criteria and their corresponding effectiveness			
Best SE cut‐off	0.375D	0.50D	0.25D
SEN (%), SPE (%)	68.8, 83.9	89.0, 71.4	69.1, 82.7
SE cut‐off when SPE ≥80%	0.375D	0.375D	0.25D
SEN (%), SPE (%)	68.8, 83.9	76.9, 82.0	69.1, 82.7

AL, axial length; AUC, area under the curve; CR, corneal curvature radius; SE, spherical equivalent; SEN, sensitivity; SPE, specificity.

Using baseline AL, AL/CR and SE to predict 4‐year incident myopia achieved AUCs of 0.585 (95% CI: 0.525–0.646), 0.740 (0.690–0.791) and 0.839 (0.797–0.881). Combining baseline SE, AL/CR, age, gender and parental myopia, the AUC increased to 0.861 (0.823–0.900) by 0.022 compared with using baseline SE alone for prediction. The ROC curve showed that the best cut‐off for prediction of 4‐year incident myopia was SE ≤0.75D, with sensitivity and specificity value of 78.6% and 75.7%. If the criterion of 80% for specificity was set, the SE was ≤0.625D to predict myopia incidence, with sensitivity and specificity of 65.2% and 82.6%.

## Discussion

The incidence of myopia was high, and the progression of refraction towards myopia was rapid in the primary school‐aged children in Baoshan District, Shanghai, similar to which was reported in Jiading District, Shanghai (SE of 0.50D towards myopia per year, and AL elongation of 0.32 mm per year).^20^ Progression of SE was 0.13D in British school‐aged children of 6–7 years old,^24^ and was 0.16D in Australian primary schoolchildren,^25^ which were much slower than in the present population of young Chinese children. Compared with children of East Asia, the present population also showed relatively high incidence and progression of myopia. In the SCORM, the annual incidence of myopia was 10.8–15.9% in children of 7–9 years old, with average annual progression of SE refraction of 0.47D.^8^ The average annual change of SE was 0.63D for myopic children and 0.29D for non‐myopic children at baseline in Hong Kong schoolchildren more than 10 years ago.^26^ The Myopia Investigation Study in Taipei reported annual progression of SE of 0.12D, 0.98D and 0.42D for children who remained non‐myopia, children who became myopia and children who were already myopia in baseline, respectively.^12^, ^27^ Compared with the RESC study carried out during 1998–2000 in Beijing Shunyi,^28^ a suburban area in mainland China, the present population showed higher incidence and larger progress of SE, probably because of the increasing severity of myopia prevalence during the recent decade, and the more urbanized setting of the present population. A recent study performed in Chong Qing also presented a relatively lower annual incidence (10.6%) of myopia and progress (0.43D),^29^ which is in accordance with the regional discrepancy of myopia prevalence in mainland China.^30^

Through classification of refractive status, we observed that the largest progression of SE and AL were possessed in persistent myopia children and the lowest progressions were in non‐myopic children. The rapid increase of SE and AL in persistent and newly developed myopia were also reported in Singaporean schoolchildren,^31^ Chinese twins^32^ and in a sample of predominantly Caucasian children.^33^ It was reported that progress of SE and AL is largest 1 year before myopia onset, and then slow down after onset.^32^, ^34^ Although it could not be verified by present study because of the 2‐year follow‐up study design, the different pattern between newly developed myopia or persistent myopia and non‐myopia might indicate different exposure of risk factors 1 or 2 years before myopia onset. In addition, as grade level increases, except for the non‐myopic children, the absolute change of SE and AL decreased. The slow‐down of SE and AL progression with age was also observed in previous literatures in both Eastern and Western schoolchildren,^24^, ^35^ which indicated that the later myopia initials, the slower the progression rate of SE and AL, and as a result, the less likely to turn highly myopia in adolescent or adulthood.

For an increase of a millimetre in AL, the amount of SE progression is larger for incipient myopia and persistent myopia (1.74 and 1.83D/mm) compared with non‐myopia (0.83D/mm) in the present population. Similar results were also found in a group of Chinese children aged 7–15 years old. Xiang *et al*. found that the ratio was about 1.25–1.60 during 2–4 years before myopia onset, increased to the highest (the ratio = 2.16) during 1 year before myopia onset, and decreased to 1.92, 2.09 and 1.60 at 1 year, 2 year and 3 year, respectively, after myopia onset.^32^ However, in the Northern Irish schoolchildren aged 6–7 years old, the ratio was 1.22, 0.45, 0.68 and 1.07 for baseline myopia, emmetropia, mild hyperopia and moderate hyperopia, respectively; for children of 12–13 years old, the ratio was even lower, which was 0.26, 0.17, 0.13 and 0.12, accordingly.^24^ Recently, Guo *et al*. reported that an increase of 1 mm in AL was associated with only 0.45D of myopic change in a group of pre‐school Chinese children with low prevalence of myopia.^36^ Despite not able to estimate lens power accurately by the present data, the variation of the ratios might be account for the differences in the amount of compensation by lens, as the CR did not change much in this age‐group of children, thus indicating an important role of lens in the onset and progression of myopia.^37^

In the analyses for possible risk factors for myopia, only parental myopia was associated with incident myopia and progression of SE, but not near work, outdoor activities or attending tutoring classes. The relationship between parental myopia and school myopia has been proposed in previous literatures.^11^, ^12^, ^13^, ^17^, ^27^ The present study found that as grade level increased, the strong association between parental myopia and progression of refraction (or incident myopia) decreased. Similar results were also observed in the Sydney Adolescent Vascular and Eye Study (SAVES), in which parental myopia was not associated with incident myopia in the older cohort.^11^ Therefore, parental myopia, probably reflecting a mixture effect of inheritance and home environment, plays an important role in early onset myopia for schoolchildren; however, gradually lose its association in the later years.

Near work and outdoor activities were two most commonly recognized risk factors for myopia^9^, ^11^, ^12^, ^16^, ^17^, ^20^; however, the present study did not observe significant relationship. One possible explanation is that the variations of time of near work and outdoor activity were too small to impact on incidence of myopia or progression of SE in the present population; therefore, positive associations could not be obtained. In addition, the questionnaires collect the average time spent on various activities per day in the most recent month, but not continuous recording the time schedule in a week or month, which could not avoid recall bias or inaccuracy while filling the investigation form. However, through analysing the eye usage time of the present study population, relatively long near work time (about 22 h per week after school and at weekend) and short outdoor activity time (about 8 h per week after school and at weekend) could probably explain the generally high myopia incident and progression in the present population. At weekend, although children spent less time on reading and writing and increased the time outdoor, the amount of increase was relatively small compared with the increase of time they spent on other near work activities such as watching television and playing computer. Therefore, publicity of the protective effect of outdoor activities in order to change the living habit of the Chinese schoolchildren is of great importance. Attending tutoring classes were not found to be associated, probably because most of the children at this grade level did not go to tutoring classes (67.8%), and among those who attended the vast majority received less than 5 h a week (68.9%). The variance might not be large enough to detect the effect of tutoring classes on myopia incidence, which was reported to be ≥5 h per week.^12^

Another finding of the study is that less hyperopic baseline refraction is a good indicator for incident myopia in the Chinese schoolchildren. Figure 3 presented incidence of myopia according to the baseline SE. Compared with Australian children in the SAVES, the distribution of baseline SE is less hyperopic in the present population; however, the pattern of incident myopia according to baseline SE categories was similar.^11^ As baseline SE become more hyperopic, the incidence of myopia decreased obviously, which is the basis that baseline SE could predict myopia onset in the future. Although several studies reported the cut‐off of baseline SE refraction to predict myopia onset in Western population (Table 6),^10^, ^11^, ^38^, ^39^, ^40^ none was reported in areas with high prevalence of myopia, such China. Children or parents could be informed the possibility of getting myopic 2 years ahead with sensitivity of 84.6% and specificity of 71%, and interventions such as increasing amount of outdoor activities and decreasing near work time could be applied. For the 4‐year prediction of myopia, using baseline SE ≤0.75D, the positive predictive value and negative predictive value were 83.4% and 69.4% based on the present prevalence of myopia. The meaning of the prediction is if a child has an SE ≤ 0.75D at the beginning of the school years, the possibility that he or she would become myopic at the end of the primary school is 83.4%. As age of onset is a crucial factor for developing high myopia later,^41^ initiation after primary school could be a potent protective factor from being high myopia in adulthood. The cut‐off points of baseline SE to predict myopia were different among literatures, probably because of different criteria for defining myopia, age at prediction, prediction period and other factors (Table 6). Generally, baseline SE turned more hyperopic, if longer prediction period is required, and turned less hyperopic, if older age at prediction is required.^10^

**Table 6:** A review of literatures that used baseline refraction to predict myopia onset

	Age at prediction	Prediction period	Cut‐off of baseline SE (definition of myopia onset)	AUC of the ROC curve	SEN	SPE	Combination factors
Hirsch^38^	School entrance	Until 13–14 years old	+0.5D (SE ≤ −0.5D)	/	59%	91%	/
Zadnik *et al*.^39^	Grade 1	Grade 8	+0.75D (SE ≤−0.75D)	0.880	86.7%	73.3%	Power of lens and cornea and axial length, AUC = 0.893
Jones‐Jordan *et al*.^40^	Grade 1	Until grade 8	+0.75D (SE ≤−0.75D)	/	62.5%	81.9%	The results were calculated after considering parental myopia.
French *et al*.^11^	6 years old	5–6 years later	Not presented (SE ≤−0.5D)	0.84			Adding outdoor activity and near work time, parental myopia, and ethnicity, AUC = 0.89
	12 years old	5–6 years later	Not presented (SE ≤−0.5D)	0.89			AUC was not improved after including the risk factors
Zadnik *et al*.^10^	Grades 1–6	Until grade 8	Grade 1: +0.75D, Grades 2 and 3: +0.5D, Grades 4 and 5: +0.25D, Grade 6: +0.00D (SE ≤ −0.75D)	0.87 to 0.93			Adding other 7 predictors, AUC ranged between 0.88 and 0.94
Our study	Grades 1–3	2 years later	+0.5D (SE ≤−0.5D)	0.862	84.6%	71.0%	Adding other risk factors AUC increased by 0.019
	Grade 1	4 years later	+0.75D (SE ≤ −0.5D)	0.839	78.6%	75.7%	Adding other risk factors AUC increased by 0.053

AUC, area under the curve; ROC, receiver operating characteristic; SE, spherical equivalent refraction; SEN, sensitivity; SPE, specificity.

The limitations of the study should also be noticed. First, 0.5% tropicamide was used for cycloplegia in the study, which might over‐estimate children's myopic status due to its relatively weak cycloplegic effect.^42^ However, 0.5% tropicamide is one of the most common cycloplegia reagent in clinical practice in Chinese hospitals, and it was proved to be effective in refractive measurement for myopia children with dark pupil.^43^ Compared with the study we did in Jiading District, Shanghai, which using cyclopentolate for pupil dilation, the changing rate of SE and AL were similar with those in the present study.^20^ Although directive comparisons of the two agents cannot be made in the present study, the difference between the two visits might offset the over‐estimation in the both visits. Second, myopia‐related risk factors were collected by filling questionnaires, which might be subject to recall bias. In addition, eye usage time in school was not involved in the questionnaires, preventing analysis of the way school type influenced myopia incidence. Moreover, the questionnaires were collected at baseline; however, children might change their behaviours in the 2 years, as shown in Figure 2, especially for those who have already been myopic, who could change their behaviours or receive treatment under the parents’ supervision. Hence, for analysing risk factors for progression of refraction and AL, only those were not myopic at baseline were included.

Incidence and progression of myopia is relatively high in the primary schoolchildren in Shanghai compared with children of Western countries, East Asia and other parts of China. Prompt and effective strategies to control myopia prevalence are in urgent need. Using baseline SE refraction ≤+0.5D could be a simple and effective indicator for 2‐year myopia prediction, and SE refraction ≤+0.75D for 4‐year prediction, enabling early interventions to control and prevent myopia.
